# Clinical features of respiratory syncytial virus bronchiolitis in an infant: rapid and fatal brain involvement

**DOI:** 10.1186/s12887-021-03045-9

**Published:** 2021-12-09

**Authors:** Paolo Bottino, Rebecca Miglino, Lisa Pastrone, Anna Maria Barbui, Giovanni Botta, Elisa Zanotto, Francesca Sidoti, Cristina Costa, Rossana Cavallo

**Affiliations:** 1 A.O.U. “Citta della Salute e della Scienza di Torino”, S.C. Microbiology and Virology U., Corso Bramante n. 88, 10126 Turin, TO Italy; 2 A.O.U. “Città della Salute e della Scienza di Torino”, Pathological anatomy and histology U., Turin, Italy

**Keywords:** RSV, Infant, Bronchiolitis, Neurotropism, Sudden infant death, Case report

## Abstract

**Background:**

Respiratory Syncytial Virus (RSV) infection is a significant cause of bronchiolitis and pneumonia, mostly responsible for hospitalization and infant death worldwide. However, in recent years the importance of extrapulmonary RSV manifestations, especially at neurological level, have become evident. Seizures, lethargy, ataxia and status epilepticus are suggestive of brain involvement, but also in their absence a direct neurological damage RSV-related need to be evaluated.

**Case presentation:**

A 40-day old male infant was admitted to the Emergency Department with severe bronchiolitis and dyspnea. The patient was reported to be coughing for a week with a vomiting episode in the previous two days. The nasopharyngeal swab confirmed the diagnosis of RSV infection and blood gas test showed hypoxemia and respiratory acidosis. For these reasons, the patient was provided with oxygen therapy. A few hours later, after an initial improvement in clinical parameters, a worsening of respiratory dynamics occurred and the patient was prepared for endotracheal intubation, but in the meantime death occurred. During all the observation period in the Emergency Room, no signs of neuropathological damage were evident. Post mortem examination showed lungs congestion with alveolar atelectasis and white matter degradation with severe edema at brain level. Microbiological analysis performed on autoptic samples confirmed the presence of RSV genome in tracheobronchial aspirate, meningeal swabs, pericardic and abdominal fluids, lung and brain biopsies.

**Conclusions:**

RSV is usually associated with respiratory diseases, however, as reported by an increasingly number of studies, the systemic dissemination of virus during severe disease can lead to a sudden infant death. The clinical picture herein reported showed a severe bronchiolitis resulting in a fatal and underestimated cerebral involvement due to RSV neurotropic behaviour and underline the need for clinicians to pay more attention to neurological involvement of RSV infection, even in absence of cerebral damage evidence.

## Background

Respiratory Syncytial Virus (RSV) infection is a significant cause of hospitalization and infant death worldwide [1]. It affects 60–70% of children by the age of 1 year and almost all children by 2 years of age and it is mostly responsible for 45–90% of episodes of bronchiolitis and 15–35% of pneumonia [2, 3]. However, in recent years the importance of extrapulmonary RSV manifestations, such as neurological, myocardial or endocrine, have become evident [4, 5].

Focusing on neuropathy, the most reported clinical signs and symptoms are seizures (reported in 7% of children and 12.9% of newborn), apnoeas, lethargy, ataxia, status epilepticus, encephalopathies and encephalitis [5–9].

The pathophysiological mechanism of encephalopathy associated with acute bronchiolitis remains undefined, but a direct invasion of the central nervous system by RSV seems to be the major mechanism responsible for cerebral involvement in RSV disease [4, 10]. As reported by Li et al. (2006), RSV can infect pulmonary neurons and interact with the chemokine receptor for CX3CL1 (CX3CR1) expressed in these cells, overrunning the Central Nervous System with resulting neurological abnormalities in patients [11].

Here, we report a case of an infant admitted with RSV-related severe bronchiolitis and a consequential rapid neurological involvement, resulting in a fatal outcome.

## Case presentation

A 40-day old male infant was admitted to the Emergency Department (ED) with bronchiolitis and dyspnea. He showed no comorbidities during pregnancy, he was born at full-term and normal.

At the time of admission, the patient was reported to be coughing for a week, worsening with a vomiting episode in the previous two days and reduction of feeding. Diuresis was regular. At the same time, he was treated with nasal and aerobic salbutamol administration. Patient’s parents also reported an older sister with phlogosis of upper respiratory tract.

The physical examination highlighted persistent dry cough, globus abdomen, marbled skin with thoracic rush, respiratory rate of 52 breaths/minute, heart rate of 160 beats/minute and arterial oxygen saturation (SaO_2_) of 93%. No sign of neuropathological damage was evident. A nasopharyngeal swab performed with molecular assay Xpert® Xpress Flu/RSV (Cepheid, USA) tested positive for RSV. No Flu A and B were detected. During observation, SaO_2_ remained 93% until one hour and half from admission in ED when, due to hypoxaemia (SaO_2_ 89%) and respiratory acidosis (pH 7.347; pCO_2_ 56.6 mmHg) (Table 1, BG1), the patient was given low-flow oxygen therapy (1 l/minute). After an initial improvement in clinical parameters (respiratory rate: 40 breaths/minute; SaO_2_ 96%), flow oxygen therapy was reduced and stabilized to 0.5 l/minute until 12 h from admission in ED. Within this time frame, SaO_2_ was observed to be settled on 96–97%. Subsequently, a sudden worsening of respiratory dynamics happened (respiratory rate: 60 breaths/minute; SaO_2_ 92%) and the therapy was changed to high-flow oxygen administration (8 l/minute). The subsequently BG2 performed after oxygen administration showed a correction in the parameters (pH 7.447; pCO_2_ 45.5 mmHg) (Table 1, BG2). Nevertheless, three hours after BG2, despite an improvement in the respiratory dynamics (respiratory rate: 48 breaths/minute; SaO_2_ 96%), a re-evaluation of the patient’s parameters was performed showing again hypercapnia and respiratory acidosis (pH 7.336; pCO_2_ 59.2 mmHg) (Table 1, BG3).

**Table 1:** Results of the laboratory tests

	BG 1	BG 2	BG 3	
Time from admission (hh:mm)	+ 01:30	+ 12:30	+ 16:00	
Temperature	37.0	37.0	37.0	°C
Ph	7.347	7.447	7.336	
pCO_2_	56.6	45.5	59.2	MmHg
pO_2_	28	39	44	MmHg
BEecf	5	7	6	Mmol/l
HCOɜ	30.8	31.4	31.7	Mmol/l
tCO_2_	32	33	33	Mmol/l
SvO_2_	48	75	75	%
HCT	40	37	31	%Pcv
HB	13.6	12.6	10.5	G/dl

*BG* Blood Gas, *pCO*_2_ partial pressure CO_2_, *pO*_2_ partial pressure O_2_, *BEecf* Base Excess in the Extracellular Fluid Compartment, *tCO*_2_ total CO_2_, *SvO*_*2*_ Venous oxygen saturation O_2_, *HCT* Hematocrit test, *HB* Hemoglobin, *PCV* packed-cell volume

**Fig. 1:**
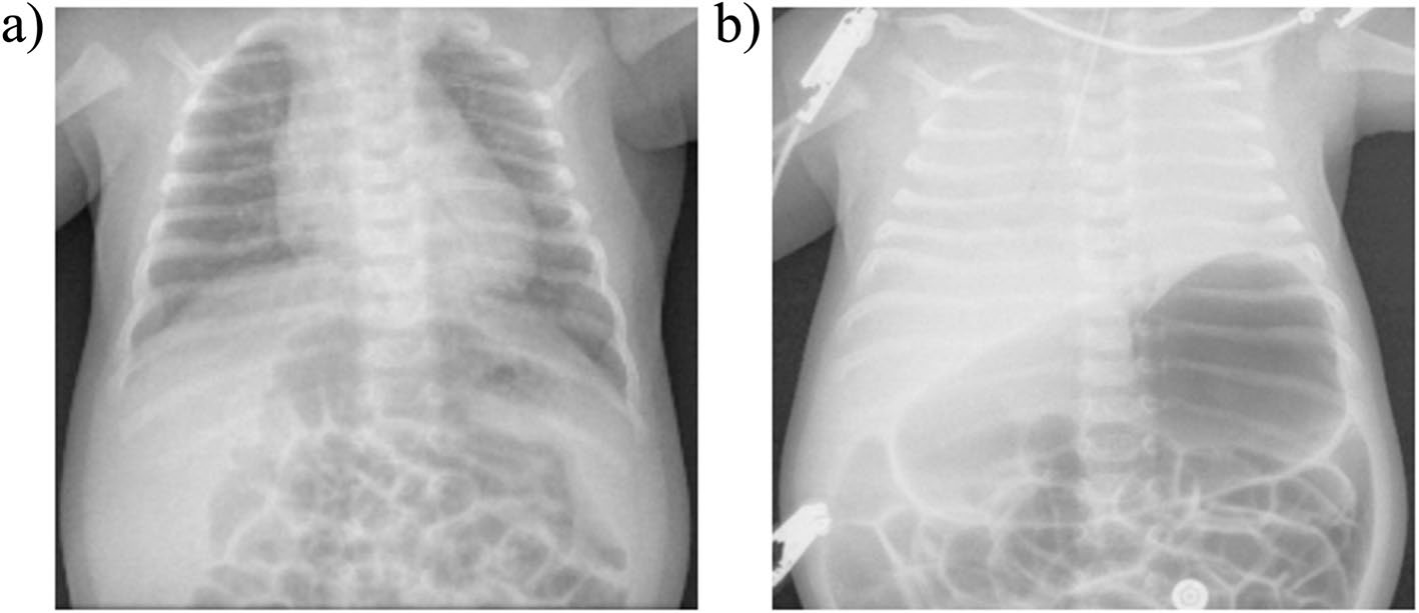
Chest Xray

Moreover, the chest Xrays performed at time of BG3 and two hours apart highlighted how fast was the clinical worsening: the first one (Fig. 1a) showed normo expanded lungs with no opacification, while the second Xray (Fig. 1b) showed diffuse bilateral pulmonary opacification with marked ectasia of the stomach.

Because of the persistent respiratory failure, the patient was treated with nebulized epinephrine and prepared for endotracheal intubation, however he died before this could be done.

Postmortem examination was carried out on the infant’s body.

**Fig. 2:**
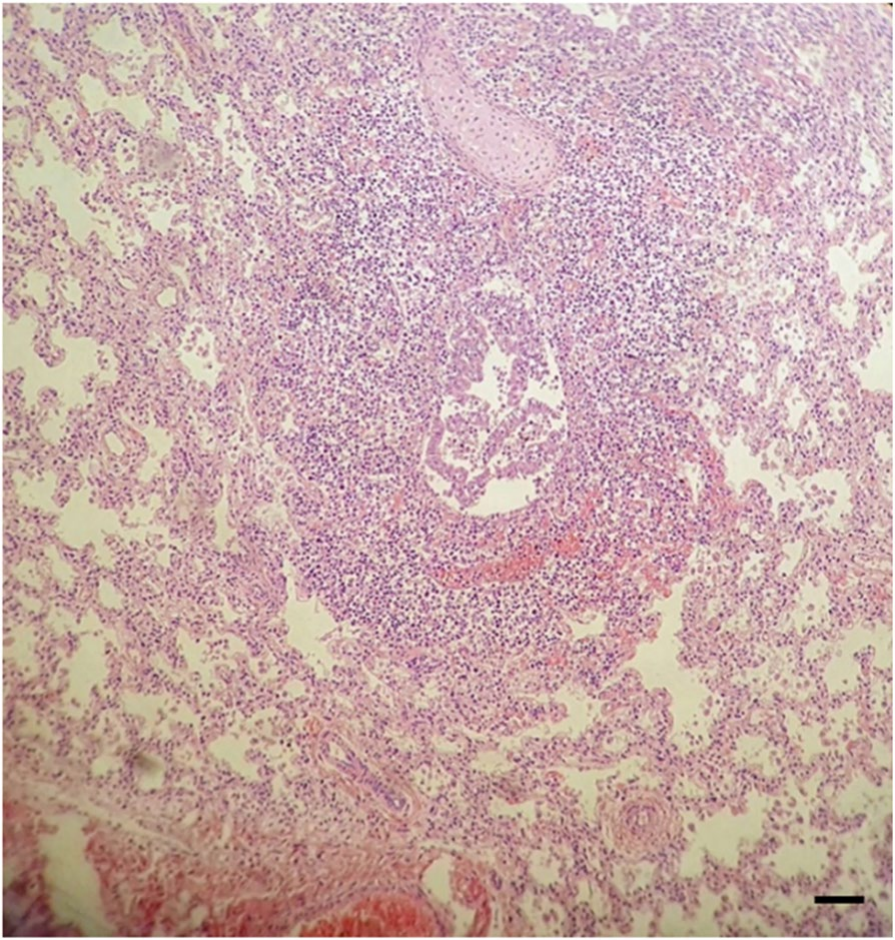
Microscopic sections of lung. 100X magnification; Leica DM 2000 Microscope with Leica DFC280 digital camera; the scale bar represents 100 μm

Lung’s microscopic examination revealed severe vascular congestion with massive bleeding, peribronchiolitis due to lymphocytic infiltrates and sporadic foci of pneumonia with reactive interstitial infiltrates and alveolar atelectasis (Fig. 2).

**Fig. 3:**
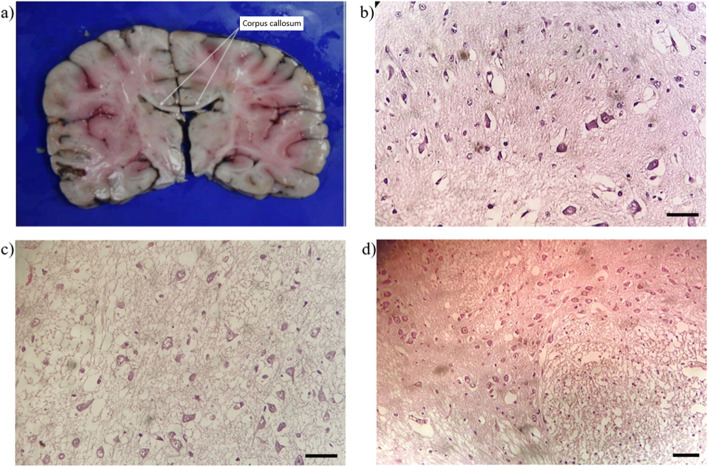
Macroscopic and microscopic brain damage. **b c** 150X magnification, **d** 125X magnification; Leica DM 2000 Microscope with Leica DFC280 digital camera; the scale bar represents 100 μm

Also, extensive injury was present in the brain, characterized by white matter degradation in the left hemisphere, basal ganglia, hippocampus, bulbar region and pons with edema (Fig. 3a).

A detailed microscopic analysis showed hypertrophic astrocytes, acutely damaged glia and focal necrosis at the bulbar level (Fig. 3b), features reported to be associated with viral infection [12]. Basal ganglia were affected by neuronal necrosis with perineuronal halo and white matter strongly damaged (Fig. 3c, d). The brain’s histological examination didn’t exclude injuries in the cardiovascular center, probably due to viral infection.

No narcotic or psychotropic drugs were detected in the blood.

Microbiological analysis performed on autoptic samples confirmed the viral etiology of brain damage and death. In fact, using molecular assay BioFire® FilmArray® RP2 Panel Plus (BioMérieux, France), RSV was detected in tracheobronchial aspirate, meningeal swabs, pericardic and abdominal fluids, lung and brain biopsies. On the contrary, the samples tested negative for Adenovirus, Coronavirus, Human Metapneumovirus, Human Rhinovirus/Enterovirus, Influenza A and B, Human parainfluenza virus, *Bordetella pertussis*, *Bordetella parapertussis*, *Chlamydophila pneumoniae* and *Mycoplasma pneumoniae*. Moreover, the microbiological cultures found neither fungal nor specific pathogenic bacteria.

Therefore, post-mortem examination revealed a severe bronchiolitis resulting in a fatal cerebral involvement due to RSV and its neurotropic behaviour.

## Discussion and conclusions

Respiratory Syncytial Virus is usually associated with respiratory diseases whose most common symptoms are fever, cough, wheezing and it typically affects children during the first two years of life. However systemic dissemination of RSV during severe disease can lead to cerebral involvement with sudden infant death [13]. The pathogenesis of RSV-related neurological damage is not yet fully understood but it has been hypothesized that RSV may enter the Central Nervous System through the hematogenous/blood-brain barrier route causing the release of several humoral neurotoxic cytokine mediators. Anyways, the direct role of RSV in inducing encephalopathy was supported by the detection of RSV antibodies or viral genome in cerebrospinal fluids (CSF) [5, 14].

Morichi et al. (2011) classified RSV-related encephalopathies into four groups (metabolic error, cytokine storm, excitotoxicity and hypoxic type) and reported that the brain imaging during RSV infection showed massive cerebral edema with subsequent diffuse brain atrophy [15]. Several other studies reported neurological complications of RSV infection, which mainly include central apnea, seizures and encephalopathy [14–17].

According to the clinical pictures described above, the index patient had an RSV infection that dramatically worsened within hours leading to cerebral edema and death. Detection of RSV genome in CSF confirmed neurological involvement.

Also noteworthy, is the finding of the virus in the pericardic fluid that could suggest a myocardial damage as probably contributing cause of death.

Unlike the previously described clinical cases, in which patients reported a confirmed RSV infection together with at least one cerebral evidence (Table 2), in our report the infant showed no neurological signs and symptoms at the time of admission. For this reason and due to the rapid worsening of the patient’s conditions, CSF sample for cytokines and nitrogen oxide determination was not collected.

**Table 2:** Clinical features of documented neurological manifestation with RSV confirmed infection at time of admission

Source, y	Age / Sex	Clinical picture at time of admission	CNS involvement at time of admission	Diagnosis of RSV infection	Outcome
Xu L et al., 2018 [14]	2 years / F	Respiratory and cardiac arrest	extensive brain edema	Antigen rapid test	Death for multiple organ failure
Morichi et al., 2011 [15]	1 year / M	CHARGE syndrome	involuntary movements, impaired consciousness	RT-LAMP	Survived with mental retardation
	11 months / F	N/A	status epilepticus, impaired consciousness	RT-LAMP	Survived with mental retardation
	3 years / M	N/A	generalized tonic-clonic seizure	RT-LAMP	Survived
	1 year / F	N/A	generalized tonic-clonic seizure	RT-LAMP	Survived
	3 years / M	N/A	Nuchal rigidity	RT-LAMP	Survived
	10 days / M	N/A	generalized tonic-clonic seizure	RT-LAMP	Survived
	4 months / M	cardiopulmonary arrest	status epilepticus, impaired consciousness	RT-LAMP	Survived with mental retardation
	27 days / M	respiratory failure	impaired consciousness, convulsions	RT-LAMP	Survived with mental retardation
	14 days / F	Apnea	lethargy	RT-LAMP	N/A
Otake et al., 2007 [16]	11 months / M	Fever, cough	generalized tonic-clonic seizure	Antigen rapid test	N/A
Zlateva et al., 2004 [17]	4 months / M	Fever, cough, tachycardia	convulsions	RT-PCR	Survived
Present report	40 days / M	Dyspnea	none	RT-PCR	Death for multiple organ failure

*N/A* data not available, *RT-LAMP* Reverse transcription loop-mediated isothermal amplification, *RT-PCR* Reverse transcriptase-polymerase chain reaction

Respiratory Syncytial Virus usually affect respiratory tract but, although rare, it can also determine a widespread organ involvement, including the brain and heart. Cerebral damage caused by RSV and perhaps concomitant cardiac involvement may led to sudden cardiac arrest in infants with bronchiolitis. Our findings, in conjunction with the before above reported cases underline the need for clinicians to pay more attention and awareness to neurological sequelae of RSV infection, even in the absence of evidence of cerebral damage.

## Data Availability

The datasets used and/or analyzed during the current study are available from the corresponding author on reasonable request.

## Abbreviations

RSV: Respiratory Syncytial Virus
CX3CR1: Chemokine receptor for CX3C
ED: Emergency Department
SaO_2_: Arterial oxygen saturation
BG: Blood Gas
pCO_2_: partial pressure CO_2_
pO_2_: partial pressure O_2_
BEecf: Base Excess in the Extracellular Fluid Compartment
tCO_2_: total CO_2_
SvO_2_: Venous oxygen saturation
HCT: Hematocrit test
HB: Hemoglobin
PCV: Packed-cell volume
CSF: Cerebrospinal fluids
RT-LAMP: Reverse transcription loop-mediated isothermal amplification
RT-PCR: Reverse transcriptase-polymerase chain reaction
